# Extracts and Gleanings

**Published:** 1871-10

**Authors:** 


SNAKE BITE CURED BY THE APPLICATION OF A COAL OF FIRE. Dr. Perkins writes to the Galveston Medical Journal:

While serving in the Confederate army I heard a very intelligent gentleman say that *firing* was the best remedy for the bite of a venomous snake. I never knew of the application of the remedy until a few days ago. A young man, aged 18 years, was bitten by a very large rattlesnake (five feet long) on the arm, above the elbow. A coal of fire was applied a short time after he was bitten. I saw him two hours after the accident, when he appeared very much prostrated, and was vomiting every few minutes—pulse very small and frequent, complaining constantly of the burn, which was pretty severe. I gave him freely of diluted alcohol. His recovery was rapid and the swelling in the arm slight.

The question now is, did the fire do any good? I think it did; not only by destroying the virus, to a considerable extent, but also, by producing a local incapacity in the veins and absorbent vessels to perform their functions. I think, in all probability, he would have died before the alcohol was given, if the fire had not been applied.

### PART II.

### Abridged Extracts and Gleanings from our Exchanges

*MULTUM IN PARVO.*

SULPHITE OF SODA IN DYSPEPSIA.—Dr. Geo. Kelner writes, that in dyspepsia, the efficiency of sulphite of soda has been marked, especially when accompanied by flatulence and eructation—these symptoms being the result, doubtless, of decomposition and fermentation of foreign material in the stomach itself, from one or more causes. It may be owing to deficiency of the gastric juice, the privation of some of its essential ingredients, or to a defective capacity of the circulatory of nervous functions (upon the integrity of which the function itself is greatly dependent); the contents of the stomach must undergo the decomposition and fermentation which invariably occur with all dead animal and all vegetable matter, when confined in vessels and subject to the continued influences of heat and moisture. To this natural operation are due the generation of gases which produce flatulence, nausea and eructation of food so frequent in dyspepsia, and also epigastric pains, diarrhœa, dysentery, colic, hepatic congestion and torpor, so frequent from the presence of foreign masses thus retained and imparting, to a greater or less extent, fatal and injurious ingredients to the blood by absorption. To arrest the process of decomposition and fermentation, I have found in my practice no means so efficient as the sulphite of soda. In such cases it acts as direct and powerful arrestor and preventive of decomposition of the food. In one case he gave the following: Cinchona comp. and cardamons with the sulphite of soda, 20 grains to the dose, in separate solutions, combining the two at the time of administering. Bathe the body once per day in a cold solution of the sulphite, and use friction freely. Reports ten days after that he feels much improved. Bowels regular; skin improved in being more moist; sleep is better and more refreshing. Continue the treatment for one month. His general appearance much improved—seems entirely well.

In several instances in which flatulence was a very prominent symptom, one or two doses of the salt appear to have immediately arrested and removed it. In all those cases of "*cardialgia*," where there seems to be an uneasy sensation at the pit of the stomach, with great heat and burning, sometimes amounting to actual pain, and which frequently extends up into the throat, with difficult breathing, vomiting, coldness of the extremities, and great restlessness and anxiety accompanying it. And in pyrosis it acts like a charm, taken after each meal. The sulphite of soda owes its virtues to the fact that it is decomposed by almost any vegetable acid, or by the hydrochloric acid of the stomach, and that this decomposition liberates sulphurous acid, which has great power to prevent alcoholic or acetous fermentation.

Another application of this salt which I consider valuable, is in the case of infants, by whom their food (the mother's breast milk) is often ejected. A dose of two to five grains of sulphite, in combination with comp. cardamon, sweetened, has proved successful in causing a retention and assimilation of the contents of the stomach when administered soon after imbibition, thus greatly promoting the health of the child. Also in cases of children where there is a fermented, swollen condition of the bowels, especially if constipated, the sulphite of soda will remove the difficulty in a short time.

I am of the opinion that the sulphite of soda, one ounce to the quart of water, makes one of the best solutions that has ever been discovered to sponge the body with in all kinds of fevers.

TINEA CAPITIS.—The following formular will be found very valuable:

| | |
|---|---|
| R.—Sodæ sulphitis, . . . | oz. ss. |
| Acid carbolici (crys), . . | dr. ss. |
| Glycerine, . . . . . | oz. iss. |
| Cerati simplicis, . . . | oz. ii.—M. |

Oinment to be used three times a day. Shave the hair from the sores and cleans them with castile soap and water; apply ointment on the parts; cover with oil-silk; give, internally, sulphite of soda in from five to twenty grain doses, in cinchona and cardamon comp., combining the two at the time of taking, three times a day; and in from three to four weeks a permanent cure will be effected.—*Dr. Kelner.*

BROMIDE OF POTASSIUM IN "SUMMER COMPLAINT OF CHILDREN." Drs. Salvater Cass and F. R. Bailey, (Knoxville, Tenn.,) recommends this salt in the above disease. The latter gentleman (Chicago Examiner,) has used it successfully, in doses of one grain and a-quarter, in mucilage, every four hours.

Chronic Hydrocephalus.—Prof. N. S. Davis, (Chicago Med. Examiner,) says: "The objects that I have attempted to accomplish by treatment in these cases has been, first to allay the morbid excitement of the cerebral structures, and second to exert a gentle, persistent, and long-continued alterative and diuretic influence, avoiding carefully any impairment of the digestive organs. I have succeeded in accomplishing these purposes by the following prescription:

| | |
|---|---|
| R.—Fl. Ex. Scutellaria, - - - | oz. ii. |
| Tinct. Digitalis, - - - - | oz. ss. |
| Iod. Potass., - - - - | dr. ij. |
| Fl. Ext. Hyoscyamus, - - - | oz. ss.—M. |

Dose—20 drops, four times a day, in sweetened water.

If the digitalis is found to be exerting too much influence, the dose must be dimished.

Mercurials are of no advantage in the chronic stage. During the early inflammatory stage, mercurials, combined with mild laxatives, might check the progress of the disease; and if promply followed by efficient doses of the iodide of potassa, any considerable effusion would be prevented, except in such cases as are complicated with tubercular deposits. If effusion does take place, and the case becomes chronic, it will be better to unite the iodide with the digitalis, scutellaria, etc., as in the formula already given.

Pill for Constipation.—Dr. Barlow, speaks in high terms of the following pills, which, for half a century, have been successfully employed in the Bath Hospital:

| | |
|---|---|
| R.—Ext. Coloc. Co., - - - - | gr. iv. |
| Calomel, - - - - - | gr. j. |

M. ft. pil. omni nocte summend.

Tinea.—No skin diseases are more annoying than those designaced by the name of "ringworms." In tinea circinata and tonsurans, one or two applications of a solution of chromic acid (one drachm to the ounce of water) has, in the writer's hands, proved very efficacious in curing the disease. Sometimes liquor potassæ painted over the affected part answers very well, or a solution of carbolic acid, which remedy checks the development of spores, and also prevents them from germinating.—*American Practitioner.*

Oil of Horse Chesnuts.—Oil of horse chesnuts prepared in France, by chemical process, is extensively used in that country as an external remedy for gout, rheumatism, etc.

PHOSPHRUS IN LOCOMOTOR ATAXIA.—Dr. Dujardin Baumetz gives phosphorus, with excellent results, in locomotor ataxia. Dr. Walter Lambert, N. Y., gave, in one case, acidi phosphorus dilut. with gratifying results. He first gave 15 drops, three times a day. In a few days he increased to 20, 25 and then to 30 minims. After ten or twelve days, he omitted the acid and gave the pyro-phosphate of iron for a week, and then returned to the acid. Electricity was also, employed every alternate day.

SPONGE TENTS.—Dr. J. B. Hough, gives the following easy method of making sponge tents:

"The sponge is first thoroughly moistened with water and pressed as dry as the strength of the hand will permit: then having formed it into the desired shape and size by the hand, or by pressing into a quill or any other tube or mould, it is immersed into the alcohol. If the spirit is sufficiently strong (90 to 100 per ct.) the sponge is *immediately* set into the given shape, which it retains perfectly after the pressure or mould is removed. It is then hard, firm and inflexible, and may be trimmed to a sharp point or any other desired shape.

To restore it to its former size and shape it is only necessary to moisten it with a few drops of water. The alcohol sets the sponge perfectly, whether the amount of compression be much or little, so that the degree of dilitation, attainable by the use of tents thus prepared, will of course, depend upon the size after moulding and the degree of pressure used. As this process of preparation works perfectly and *without delay*, its advantages are obvious. A small quantity of carbolic acid added to the alcohol would render the sponge antisceptic.—*Boston Medical and Surgical Journal.*

POWDER for excessive perspiration of the hands and feet:

| | |
|---|---|
| Carbolic Acid, - - - - | 1 part. |
| Burnt Alum, - - - - - | 4 parts. |
| Starch, - - - - - | 200 parts. |
| French Chalk, - - - - - | 50 parts. |
| Oil of Lemon, - - - - | 2 parts. |

Make a fine powder, to be applied to the hands and feet, or be sprinkled inside of gloves or stockings.—*Pharmaceutische Centralhalle.*

BURNS, AND THEIR TREATMENT.—Having tried everything of which I could hear, I have settled down to the conviction that olive oil and the white of eggs, in equal quanties, beaten up thoroughly together, and applied with a soft brush or feather, is the best.—*Dr. Jackson on Medical Expedients.*

Essence of Turpentine in the Treatment of Wounds.—It is stated in Archiv. Med. Belges, that essence of turpentine has lately proved the only successful measure against the progress of hospital gangrene. All other substances were tried in vain. Thus the wounds being well washed, they were dressed with lint steeped in essence of turpentine, and they very rapidly became healthy and healed.—*Practitioner.*

Therapeutic Value of Scutelaria.—Dr. D. C. Leavenworth (Med. and Surg. Reporter), says: "The value of scullcap consists in its combination of nervine tonic and other medicinal qualities. I have used it in various nervous diseases—more particularly in those nervous diseases—which require a nervine and tonic combined. I consider it especially applicable in low forms of fever, where the patient is quite restless and nervous. Also in the various forms of nervous disease incident to females. I have obtained marked success from its use in cases of epilepsy, infantile cerebral diseases, mental diseases resulting from masturbation. It will be found beneficial for calming the excitability of the nervous system, especially where the brain has been overtaxed, and the mind is depressed and weakened, referable to cases and anxieties.

Hooping Cough.—Dr. A. J. Haile, of Philadelphia, says: As to the remedies that have been so highly recommened by different authors as specifics for hooping cough, I can only say that they are quite various, and a great many that should not be relied on. However, there is one that I do not hesitate to recommend as being one of the best that probably has ever been introduced for this malady, it being composed of the following:

| | |
|---|---|
| R.—Bromide potass., . . . | dr. i. |
| Bromide ammonia, . . . | dr iii. |
| Iodide potass., . . . . . | dr. i. |
| Bicarbonate potass., . . . | dr. ii. |
| Fluid ext. black cohosh, | |
| Glycerine, . . . . . | aa. oz. iii.—M. |

S.—A teaspoonful at bed time. The above remedy I have used quite extensively, with the best beneficial results.—*Eclectic Medical Journal.*

Cholera Infantum.—Dr. Galloupe, of Lynn, Mass. (Boston Med. and Surg. Journal), has employed, with good success, the following combination as diet in cases of cholera infantum: cream, one tablespoonful; water, four tablespoonfuls; lime water, one tablespoonful.—*Medical Record.*

TREATMENT OF ENLARGED TONSILS.—A writer in the British Medical Journal, states that in chronic cases of enlarged tonsils he has never seen any permanent good from incision, but has obtained the best results from alum, either as a gargle or in powder, applied with a damp brush, or as a dust of equal parts of burnt alum and gum arabic, blown upon the parts with an india rubber bottle. The applications require to be persevered in. Another physician thinks the sulphate of pottassa, in doses of from five to fifteen grains, given every morning for five or six weeks, almost a specific for enlarged tonsils in children. Should the remedy produce purging, the dose should be lessened.—*Am. Practitioner.*

OINTMENT FOR HÆMORRHOIDS.—The following by the late Prof. W. R. Fisher:

| | | |
|---|---|---|
| R.—Sulphate of Morphia, | . . | gr. iii. |
| Extract of Stramonium, | . . | gr. xxx. |
| Olive Oil, | . . . . | gr. lx. |
| Carbonate of Lead, | . . . | gr. lx. |
| Lard Cerate, | . . . | dr. iii. |

Rub the extract, if not uniformly soft, with a few drops of water; add the powders and olive oil, and rub till perfectly smooth, and then incorporate them with the cerate.—*American Journal of Pharmacy.*

TREATMENT OF CHRONIC DIARRHŒA AND DPSENTERY.—Dr. J. C. Whitehill, (Medical Archives,) speaks highly of the following method of treating these diseases, suggested to him by Dr. York:

"Take of the *inner* bark of the pine tree, from a-half to one ounce; boiling water, O i.; loaf sugar, q. s. Take this amount of tea daily until relieved."

GONORRHŒA.—Davis L. Field, M. D., of Jeffersonville, In., writes that he has very marked success in the treatment of this affection by the use of the following; Bromide of potash, two ounces; water, four ounces. Dissolve, and add tinct. iodine, three drachms. Of this he gives a tea-spoonful every four hours. If it produces headache he uses the medicine less frequently, but continues it until the discharge is arrested, which he finds to be usually within four or five days. He administers a saline cathartic every morning, and directs weak injections of sulphate of zinc —one drachm to a pint of water—Three or four times a day; and insures cleanliness of the parts by having them bathed often in cold water. He has not found his patients to suffer from chordee, or be troubled with venereal desire, while on this treatment.—*Am. Practitioner.*

LOCAL ANÆSTHESIA.—Dr. Spessa states, in the Bulletin, that he has succeeded in preventing pain, during the slitting of a fistulous tract, by injecting a solution of morphia into the tract before the use of the knife. The same author had occasion to touch the vulvar vegetations of a girl with butter of antimony; the pain was very acute, but disappeared on the part being brushed over with a solutiou of morphia. A boy of fifteen, suffering from hip-joint disease, required an issue over and behind the great trochanter. An injection of morphia was first made over the region, and Vienna paste applied, which latter remained about eight minutes. The paste did not give any pain. Dr. Spessa states that he would be glad to hear that a fair trial has been given to this mode of using morphia.—*Lancet.*

POTION FOR THE FIRST SYMPTOMS OF CHOLERA.—*By Delioux.*

| | | |
|---|---|---|
| Ether, - - - - | 1 | drachm. |
| Ext. of Rhatany, - - - | 1 | " |
| Syrup of Opium, - - | 1 | ounce. |
| Hydrolate of Peppermint, - | 2 | " |
| Hydrolate of Orange Leaves, | 2 | " |

Mix the qydrolates, dissolve into them the extract of rhatany; add the syrup of opium, and lastly, the ether.

Dose—A spoonful every quarter of an hour, in the beginning, then diminishing as the patient grows better.—*Bull. de Ther.*

PHOSPHORUS PILLS.—Dr. Radcliffe, having tried various means of administering phosphorus, has at length succeeded in effecting this in the form of pills; and as other medical men are now ordering phosphorus in this form, we thought it desirable to publish the formula for the information of our readers. Take of phosphorus six grains, suet six hundred grains, melt the suet in a stopped bottle, capable of holding twice the quantity indicated; put in the phosphorus, and when liquid, agitate the mixture until it becomes solid; roll into three-grain pills, and cover with gelatine. Each pill will contain one-thirty-third of a grain of phosphorus.—*Pharmaceutical Journal.*

PEPSIN IN THE VOMITING OF PREGNANCY.—A number of French physicians declare the efficacy of pepsin in the vomiting of pregnancy. It should be given in the dose of eight or ten grains before eating. Hydrochloric acid is also recommended as equally effieient—30 to 60 drops to be taken daily, properly diluted. Strychnia, we think, is not inferior to either—the 20th to the 12th of a grain three times a day.

Iodoform in some Phases of Syphilis.—Dr. J. H. Davenport (Boston Medical and Surgical Journal), speaks favorably of the value of iodoform in syphilitic ulcers. He reports a number of cases. In one patient he counted sixty ulcers on the legs, and thirty on the arms and face—mostly of large size. On the nates two sores two inches in diameter, and one on the wrist two inches broad and nearly encircling it. To this patient he gave—

R.—Iodoform, . . . . . . gr. ij.
Sulph. Quinine, . . . . . . gr. i.—M.

S.—Take three times daily.

Also, Iodoform ointment (gr. xxx to oz. i,) was applied to the sores. A marked influence was observed at once.

In speaking of this drug generally, he says: "Iodoform is found in the shops as a light yellow powder of small, pearly crystalized scales. It is used both externally and internally; externally as an ointment: R.—Iodoform, grs. xxx.–lx.; simple cerate and lard, each one half oz.—M. Or, better still, is simply dusted upon the surface and a rag smeared with cerate or a bit of lint dipped in glycerine placed above; internally, the best form of administration is in pills containing two or three grains each of iodoform. The power of iodoform is greatly enhanced by adding to these pills Valleix's iron, which is protected from combining with the iodoform by the insolubility of the iodoform in water. These pills, if preferred, may be sugar-coated.

Giving in overdoses, iodoform causes, says Ringer, a species of intoxication, succeeded by convulsions, with tetanic spasms. It imparts its peculiar odor to the breath of the patient, a fact which the writer has often noticed on entering a room in which patients taking the drug have been lying.

Chronic Otorrhœa.—The use of the following lotion is productive of almost uniformly good results in chronic otorrhœa: Carbolic acid, one drachm; glycerine, one ounce; distilled water, five ounces.—*Mr. J. P. Pennefeather.*

Electricity in Tic Doloreaux and Hemicrania.—Dr. Berger reports twenty-five cases of tic doloreaux treated with the galvanic current—the positive pole wet and applied to the affected parts, the negative on the knee or in the hand. But when the pain was only at one point, the headed electrode was employed. The current was moderate, lasting five to eight minutes daily. After ten to twenty sittings, twenty-two cases cured.—*Indian Jour. of Med.*

New Treatment for Smallpox.—Dr. J. J. Garth Wilkinson, of London, England, has called the attention of the medical world to a new method of treating smallpox, which he has tried in four cases of varied degrees of violence, with complete success. In these cases he used *hydrastis canadensis* and *veratrum viride* both internally and locally as a lotion. The former, he says, extinguishes the varioloid poison, while the latter subdues the inflammation and primary fever. With regard to diet, he advises a judi. cious use of brandy and water, claret, Carlowitz or Hungarian wines (port when the patient has begun to amend), beef-tea and (in convalescence) fruit.

Hæmorrhoids.—In recent cases of hæmorrhoids, Prof. Nathan R. Smith, of Baltimore, advocates this formula:

| | | |
|---|---|---|
| R.—Sulphur lot., | - - - - | oz. j. |
| Fol. sennæ pulv., | - - - - | dr. j. |
| Ol. fœnic, | - - - - - | gtt. xx. |

M. ut. f. pulv. S.—Give a heaping teaspoonful every other night. The parts must be bathed three or four times a day with cold water, and especially after stool.—*Nashville Journal of Med. and Surgery.*

Valuable Combination.—A correspondent, Dr. C. W. Davis, of Iowa, writes: "Sulphate of zinc and chlorate of potass. *intimately ground* together in equal quantities, will be found a most excellent and satisfactory remedy. The various forms of stomatitis, sore throat, ophthalmic, and many cutaneous diseases, yield at once, by its use. I was led to make this combination to economize space in my vial case. Instead of carrying both the zinc and potash, I carry the combination. Even in internal administration the zinc adds to the efficiency of the chlorate.

Ten grains to the ounce of water as a collyrium. One drachm to four ounces of water as a wash in stomatitis, and gargle in sore throat, to be used stronger if necessary as a solution. The zinc and potash must be *most* intimately ground together to an impalpable powder to insure its perfect and certain efficacy. From the efficiency and curative power of this combination there is evidently a *physiological* affinity."—*Medical and Surgical Reporter.*

For Morning Sickness.—R. Tinct. Nux Vomica, gtts. x.; Water, oz. iv.; dose a teaspoonful. At first, and when the nausea is persistent, it may be repeated every half hour; as it is relieved, increase the distance between the doses, until finally it is given three or four times a day.—*Dr. J. M. Scudder.*

TREATMENT OF ECZEMA.—Dr. Erasmus Wilson, F. R. S. (Journal of Cutaneous Medicine), after endeavoring to show, in the treatment of eczema, that the patient needs repose, quiet, rest and proper and nutritous food without stint or limit, asks: "What else is to be done? Shall we purge or physic the patient? God forbid. If the bowels are confined, help them with a little magnegia and rhubarb, or manna, or castor oil; if the patient is emaciated, and exhibits a tendency to waste, the prostration may be checked by cod liver oil; but the one and almost indispensable remedy is the nerve-restorer, arsenic. The two minims of the liquor arsenicalis three times a day, in the following combination:

| | | |
|---|---|---|
| R.—Vini Ferri, | . . . . | oz. iss. |
| Syrup Simplicis, | . . . . | dr iij. |
| Liquoris Arsenicalis, | . . . | dr. i. |
| Aquæ Anethi, | . . . . | oz. ij.—M. |

One drachm will give two minims of liquor arsenicalis. It may be administered pure, and just at the end of the meal, so as to be mixed with the meal in the stomach. The physician should watch narrowly the arsenical solution to see that it occasions no nausea, no gripes, and no prostration of power. These are its primary effects when it disagrees with the system, and there are sundry subsequent effects, such as a puffy swelling of the eyelids, the cheeks, or limbs; and later still, congestion of the vessels of the conjunctiva.

ANTIZIMOTIC TREATMENT OF SYPHILIS.—Prof Morgan, (Dublin Quarterly Journal Medical Science,) recommends creosote in cases of syphilis where mercury is contra-indicated, and says, the earlier the stage of eruption or other manifestation the more favorable will it be for this mode of treatment. In some instances of roseola the effect was very rapid and decisive. The formula, Mr. M. found convenient, was—

| | | |
|---|---|---|
| R.—Creosote, | . . . . . | f. dr. ss. |
| Mucilage, | . . . . . . | f. oz. j. |
| Laudanum, | . . . . . | f. dr. ss. |
| Peppermint-water, | . . . . | f. oz. vij.—M. |

Dose—half an ounce four times a day.

Use at the same time a warm bath every second night containing two or three drachms of carbolic acid.

*Syphilis in Children.*—So long as the affection is confined to the surface, and there is no internal deposits, mercury, gently used, acts almost as a specific, and without any ill effects whatever. Chlorate of potash has succeeded in no case, creosote in some.—*American Practitoner.*

Bromide of Potassium and Fluid Extract Gelseminum in Headache.—Dr. T. M. Woodson, of Tenn., a practioner of very large experience, writes: "I was deeply interested in the article on bromide of potassium in headache, in the February number of the American Practitioner. I well remember how the author used to suffer. I too have myself been almost as great a suffer as he was. For a year past, however, I have derived great relief from the bromide of potassium, more indeed than from any single remedy; but I have ususally taken it along with the fluid extract of gelseminum. These remedies, in combination, have seemed to me of especial efficacy in the neuralgic headaches of females, particularly where opium was not borne well."—*American Practitioner.*

Ergot to Arrest Excessive Perspiration.—Dr. Christman, it is stated, in the Wurtemb. Med. Corresp. Blatt, No. 20, has seen in many cases of excessive perspiration, a diminution of the cutaneous discharge ensue promptly upon the exhibition of ergot (8 to 10 grammes four times a day)—Centralblatt f. d. Med. Wissenchaften.—*Medical Journal of Medical Sciences*, April 1870.

Chloral in Senile Gangrene.—Dr. J. C. Butcher, North Lewisburg, Ohio (Leavenworth Med. Herald), publishes a case of senile gangrene, in which the administration of chloral had a magical effect in giving rest to the patient. Previously, morphia was given by hypodermic injections, and vomiting followed the next day after its administration. He finds that small doses of chloral produce the same effect as large ones. His formula is as follows:

R.—Chloral, . . . . . . dr. i.
Simple Syrup, . . . . . oz. j.—M.
Dose—a teaspoonful every hour.—*Medical Record.*

Raw Beef in the Vomiting of Pregnancy.—Dr. James L. Bailey, reports the case of a patient, three months advanced in pregnancy, who was unable to retain anything she had eaten during three days. Believing this condition due to "reflex action," he ordered his patient to take teaspoonful-doses of raw beef, chopped fine, at intervals of three hours, with a little cayenne pepper and salt sprinkled upon it. The dose was repulsive at first, but after the second teaspoonful the vomiting ceased, and during the day the nausea disappeared. She rapidly gained flesh and the sickness was permanently relieved.

Mammary Abscess.—It is said that enveloping an indurated mamma with oil-silk will prevent the development of an abscess.

Tansy in Epistaxis.—Dr. C. P. Uhle, (Med. Reportor,) highly recommends the *tanacetum vulgare* as a remedy in epistaxis. He suffered freqently from this affection while a student, and upon one occasion accidentally plucked a leaf of tansy and applied it to the part. The hæmorrhage ceased immediately, and the remedy has never failed since. Subsequently, numerous experiements were made by Dr. Uhle, with entire and gratifying success. In some instances, the simple aroma or odor of the plant was sufficient to quell the most active hæmorrhage.

Diphtheria.—Dr. James E. Reeves, Wheeling, West Virginia (The Med. Times), states that experience has abundantly satisfied him that the use of strong caustics is not advisable in the treatment of diphtheria—that in numerous instances they aggravate all the symptoms, and thus greatly endanger the patient. The tincture ferri chloridi may be applied in full strength to the diphtheritic patches and to the surface around their borders, by means of a camel's hair-pencil; but even this practice he has generally abandoned during the last five or six years, and now contents himself with gargles or the atomized spray of the chlorine and iron mixture every hour or two when the patient is awake.

| | |
|---|---|
| R.—Potass. Chlorat, . . . | dr. ij. |
| Acid Hydrochloric Puri, . . | dr. iss. |
| Aquæ, . . . . . | oz. vij. |
| Tinct. Ferri Chloridi, . . . | oz. j.—M. |

At the same time this mixture may be internally administered, in the dose of from twenty drops to a teaspoonful, with or without a little simple syrup, every two or three hours, with the addition, if need be, of sulphate of quinia. Warm atomized inhalations of the chlorine and iron mixture, according to the above formula, promise, he thinks, the greatest hope in cases of dipththeritic croup. —*Med. Record.*

Treatment of Hydrocele by Injection of Chloroform and Compound Tincture of Iodine.—Dr. Moses Barrett (Trans. Wis. State Med. Soc.) reports a case of hydrocele of the left testicle, in which about 4 oz. of fluid were drawn off, and the following injected into the sac: R.—Chloroform and comp. tinct. iod., of each 1 drachm; aquæ, ad 1 oz. This was allowed to remain between three and four minutes. Sharp pain in the scrotum and back was experienced, with nausea and faintness. There was, for a few days, some swelling and tenderness of the scrotum, which was relieved with a lotion of acet. plumb. The recovery was perfect.—*Medical Record.*

Iodo-Bromide of Calcicum Compound.—This new remedy, prepared by Tilden & Co., New Lebanon, N. Y., is attracting attention. Dr. Rogers gave the "Elixir" in a case of "Scrofulous diathesis," attended by profuse suppuration from abscess, with great success after using all the standard remedies in vain. Dr. S. H. Potter, used it successfully in a case of cancroid tumor of of the rectum. Dr. J. H. Haynes, regards it a very valuable discutient in glandular enlargement. It has also in other hands, relieved neuralgia, lumbago, ottorrhœa and hemicrania. Chronic rheumatism has been almost instantly relieved by it—banishing pain whenever it recurs. A weak solution removes pimples on the face. The following formula will be found very valuable in *itch*:

R.—Iodo-Bromide of Calcicum Comp., oz. i.
Water, - - - - - - oz. iv.—M.

S.—Sponge parts effected twice daily. Suspend if it produce too much irritation—to renew if necessary—graduating strength to suit.

Chronic Diarrhœas of Children.—In the form known as lientaria, in which there is such a condition of the intestinal canal, that the food, quickly after ingestion, is expelled only superficially digested, Muller says, nux vomica may be regarded as a specific, while opium is quite useless.

Nitrate of Silver is indicated in diarrhœas under the following conditions: 1. Croupous deposits in the mouth and fuaces, such as frequently complicate chroninc intestinal catarrh. 2. A peculiar redress and smoothness of the tongue. 3. Irrepressible thirst.

Formula for Ammenorrhœa.

R.—Tincture polygoni punctati saturatæ, oz. iss.
Tincturæ ferri chloride, - - oz. ss.

Misce et signa: A teaspoonful thrice daily. Diet to be generous; gentle horseback exercise and the lumbo sacral douche every night before retiring.

Prof. Eberle is quoted, as having reported twenty cases of ammenorrhœa and dysmenorrhœa which were treated with this remedy alone, with complete success.

A "Specific" for Asthma.—R.—Tincture Ptelea (or Fluid Extract), oz. ss.; Simple Syrup, oz. iijss. A teaspoonful every three or four hours.—*Dr. Scudder.*

Croton Tig.—In eczema, paticularly of the scrotum, and in herpes circinatus, there is no remedy, to my knowledge, like it.

TREATMENT OF CHILBLAINS.—For this prevalent and often painful malady, a perfectly safe and effectual remedy is, Mr. Skey says, obtainable in the employment of laudanum, taken internally in very small doses, of from two drops for young children night and morning, up to six or eight for adults. It is in such quantities perfectly harmless, and, as a rule, will effect a cure in the course of four or five days.

Dr. George P. Rugg, in a letter to the Lancet, states that he has found an excellent remedy in the new disinfectant "chloralum," for unbroken chilblaims. It should be applied undiluted night and morning, using a moderate amount of friction.

The following recipes appeare in the Pharmaceutical Journal, of November 26th, and December 17th:

| | |
|---|---|
| R.—Glycerine, . . . . . | oz. iij. |
| Tinct. of Arnica, . . . . . | oz. j. |
| Spirit of Camphor, . . . . | oz. ss. |
| Rose-water, . . . . | dr. j. |

To be well rubbed in, night and morning.

DR. DEWAR'S LOTION.—

| | |
|---|---|
| R.—Sulphurous Acid, Glycerine, aa, . | oz. j. |
| Distilled Water, . . . . | oz. ij. |

| | |
|---|---|
| R.—Lin. Belladonna, . . . | dr. ij. |
| Lin. Aconiti, . . . . . . | dr. j. |
| Acid. Carbolic, . . . . . | m. x. |
| Collodion flexile, . . . | ad oz. j.—M. |

Apply with a camel-hair brush.

The above is for unbroken chilblains; if they are broken, the lin. aconite is to be omitted.—*Lancet.*

DYSMENORRHŒA.—Dr. J. M. Scudder, says, he has employed the following, four years without a failure:

| | |
|---|---|
| R.—Tinct. Pulsatilla, Tinct. Macrotys, | aa. dr. j. |
| Water, . . . . . . | oz. iv. |

A teaspoonful every four hours. Commencing three or four days before the expected period, it is continued until the discharge is free and painless. And it is thus repeated from month to month until a cure is effected. Any treatment that may seem necessary to restore the general health, should be carried on during the inter-menstrual period.

WASH FOR SORE NIPPLES.—The best application which I have found for sore or cracked nipples is *benzoic acid* in solution.

FLATULENC—ITS TREATMENT.—Dr. John Chapman, states, (Med. Press and Circular,) "There are, fortunately, two plans, both often remarkably successful, of relieving the distress caused by gaseous distension, without resorting to drugs at all, and therefore without submitting to the evils sometimes inseparable from their employment: either the prolonged use of the warm bath, at 100° Fahr., or the judicious application of the spinal ice-bag often operates like a charm in respect to both the rapidity and the completeness with which the gaseous swelling is made to subside*; and, in my opinion, experience will prove to every physician who gives these methods an adequate trial, that very few cases will be met with in which effectual relief will not be given by resorting either to one of these experiments or to both of them in succession."

| | | |
|---|---|---|
| DEPIATORY POWDER.—Quicklime, | - | gram. xxx. |
| Yellow sulphate of arsenic, | - - | gram. ii. 50 cent. |
| Starch in powder, | - - - | gram. xxiv. 00 " |

Mix.—To use this powder, it is only necessary to dissolve it in a small quantity of water, and apply it to the spot from which we desire to remove the hair. From one to two minutes are sufficient to produce this result.—*Repertoire Pharmaceutique.*

SPERMATORRHŒA.—We must bear in mind that we have two special conditions to meet; the one where there is a discharge of the prostatic fluid, and the other accompanied by an emission of sperm.

For the abnormal or enfeebled condition of the prostrate gland the tincture of iron, given in thirty-drop doses, is a specific. As regards its *modus operandi* it exerts a specific influence over the urinary organs, allaying any inflammation of the prostate and adjacent structures ; it acts to a considerable extent upon the blood, by which it improves the quantity and quality of that vital fluid, promotes digestion, and favors assimilation. It should be given at least as often as four times a day to obtain its specific action.

For spermatorrhœa proper Lupulin is the remedy given in fifteen grain doses. It acts very much upon the same principle that opium does, except it leaves no nausea, headache, tumors, or other symptoms of diminished or irregular nervous action. Besides it possesses no astringent properties that would render its use impracticable in the treatment of this disease. I usually prescribe Lupulin in the doses before mention, four times a day, the last dose to be taken on going to bed.—*Eclectic Medical Journal.*

Sulphate of Bebeeria in Chronic Inflammation of the Uterus.—A writer in the American Practitioner, having had his attention directed to Dr. Merrill's article upon "Uterine Diseases" in this Joural, in which he advocated bebeeria as a deobstruent in these disorders, was induced to prescribe it in a case of disordered menstruation, with profuse leucorrhœa in the intermenstrual periods. At first he gave three grains of the sulphate of bebeeria, with one-eighth of a grain of the extract of *cannabis sativa*, three times a day—subsequently the quantity of bebeeria in each dose was increased to four grains, of hemp, to half a grain. This treatment was commenced just after a very painful menstruation. The next period was almost painless, the patient not finding it necessary to remain in bed during it. The improvement up to the present time has been constant, and he has every reason to believe it will be permanent. The treatment was continued for two months.—*Medical Record.*

New Remedy for Dysmenorrhœa and Tenesmus of Dysentery.—Prof. W. K. Bowling, Nashville Medical Journal, reports the following case:

Mrs. H——, aged twenty-two years, married, suffers horribly with dysmenorrhœa. Has never been pregnant, and never suffered in her turns before marriage; is a delicate, educated woman. Desiring me to prescribe for her, I did so, as follows:

℞.—Gum opii, Gum camphor, . aa. gr. vj.

Lupulin, . . . . . gr. xxxvj.

M.—and make 12 pills.

Begin upon the day before the expected turn, and take one pill every six hours.

She took the medicine, and passed her menstrual period, as I had promised her, with a full flow, and without any pain. But the intolerable opiate so worried her, that, at her next period, she preferred the pain to the medicine, and would not take it. She continued to suffer at each period for several months. She then, at the very beginning of the pain, sat a few moments on a chamber, in which a dr. j. of spirit of ammonia had been placed. In a minute the pain was gone, and did not return. The same result follows at each period since.

Dysentery now, August 20th, is almost epidemic here. We have tried the ammonia in a great number of cases for the tenesmus, and always with gratifying effects.

---

The Georgia Medical Companion should be taken by every physician in the land.

*Carbolic Acid in Children's Diseases.*—Dr. N. S. Davis gives the following experience in the Chicago Medical Examiner:

"During the last two years, we have prescribed the carbolic acid very often, and in a considerable number of morbid conditions. In the various grades of irritation or morbid sensitiveness of the mucous membranes of the alimentary canal, especially in children, we have found it a very valuable remedy. A few cases will serve to illustrate more fully the application of the remedy than we could convey in any other manner.

*Case* 1.—A. B., child eight months old, nursing. The bowels had been slightly loose for three or four days, the discharges thinner and more offensive than natural, but not more than three times a day, until July 3d, 1870, when it began to have active diarrhœa, the discharges being very thin and of a greenish color, accompanied by a prompt rejection of whatever it took into its stomach, either by nursing or drinking. It was not the active vomiting of severe cholera morbus, but that morbid sensitive of the stomach that causes rejection of the ingesta and serous diarrhœa. There was no febrile reaction, but rather paleness and coolness of the surface. The mother was directed to let the child nurse often, but only a little at a time, and give it no drinks except one or two teaspoonsful at a time of cold water and mucilage; and the following prescription was given:

| | | |
|---|---|---|
| R.—Carbolic acid crystals, - - | grs. | iij. |
| Glycerine, - . - - - | oz. | ss. |
| Camp. tincture opii, - - - | oz. | ss. |
| Water, - - - - - - | oz. | ij.—M. |

And give 20 drops every two hours until the stomach and bowels are quiet.

When there have been no evacuations up or down for twelve hours, then extend the intervals between the doses to three hours. Under this treatment the vomiting ceased during the first twelve hours, but moderate diarrhœa continued, and the medicine was also continued at intervals of three hours. On the third day commencing the treatment there was no vomiting and only two intestinal evacuations more healthy in character. The same medicine was continued four times a day, for three days, when the child appeared well and treatment was discontinued. During the summer of 1870 we treated more than 70 cases similar to the one just related, embracing children from six months to two years of age, with the same formulæ, and nine out of ten speedily recovered. Such of the children as had been weaned were fed on small but frequently repeated doses of a thin porridge, made of sweet milk and wheat flour. In a few instances the medicine appeared to exert no influence over either the vomiting or diarrhœa, and other remedies were made available. It will be remembered that the cases here alluded to were recent and simple in their nature. The following will illustrate another class of cases of greater severity, and of very frequent occurrence during the months of July, August and September.

*Case* 2—*July* 27*th.*—Called to C. T——'s child, aged 15 months, still nursing. The child had commenced to have moderate diarrhœa, or, "summer complaint," as it is termed, during the first week in July, which had continued, with only occasional vomiting when it took too much into its stomach, until the 24th. It had become pale and thin in flesh, but still most of the time cheerful, and the mother, as is usual in such cases, attributing the looseness to "teething," had used no remedies except one or two doses of castor-oil. During the night of the 24th the child became more restless, the bowels moving every two or three hours, and the stomach promptly rejecting whatever was taken into it. The intestinal discharges were very thin, yellow, and offensive. The following day a physician was called, who prescribed suitable doses of anodyne and alterative powders, mustard sinapisms over the epigastrium, and the next day some laxative mixture, sufficient to move the bowels. Almost every dose of medicine, however, was rejected by vomiting, and the original symptoms continued without abatement. When we were called on the 27th, the child was much emaciated, the countenance haggard, extremities cool, pulse quick and feeble, paroxysms of great restlessness, with intervening somnolency—almost every paroxysm of restlessness ending in a discharge from the bowels of a greenish yellor color, and almost as thin as water, with little specs of mucus in it. There was pretty uniform vomiting within a few moments after nursing or taking any kind of drink. The urinary secretion was very scanty. We advised the mother to let the child nurse only a little at a time, but often, and to give no other drink except teaspoonful-doses of ice-water, of which it was very fond. For medicine we directed the following:

| | | |
|---|---|---|
| R.—Carbolic acid crystals, | - | grs. iii. |
| Glycerine (pure), | - - | oz. ss. |
| Water, | - - - | oz. ijss.—M. |

And give half a teaspoonful every hour until the vomiting ceases, and the breast milk is retained well.

Also the following:

| | | |
|---|---|---|
| R.—Nitrous ether, | - - - - | oz. ss. |
| Camp. tincture opii, | - - - | oz. ss.—M. |

Give 20 drops in half tablespoonful of sweetened water, every three hours, to help allay the irritability of the bowels, and promote more active secretions of the kidneys.

*July 28th.*—The vomiting has nearly ceased; the evacutions from the bowels are less frequent, but nearly the same in character, and the urine only slightly increased in quantity. Ordered both prescriptions continued, but the solution of carbolic acid only every three hours, making it come alternatively with the paregoric and spirits of nitre.

*July 29th.*—Child nurses well, and retains all it takes into its stomach; countenance much improved; urine more abundant; but the intestinal discharges continue to occur every three or four hours, and remain thin and pretty copious. Directed the carbolic acid solution to be continued every six hours, and half-way between, one of the following powders, viz:

R.—Sub. nit. bismuth, - - - grs. xii.
Pulv. geranium root, - - - grs. iv.
Pulv. doveri, - - - - - gr. i.—M.

Divide into six powders.

Under this treatment the bowels steadily improved, and on the 1st of August the carbolic acid was omitted, and only one powder given each night and morning; and after three days more they were dispensed with altogether, the child needing no further treatment. As already remarked, this case is the representative of a large number that were treated, and in nearly all of which the carbolic acid was of great service in allaying the gastric irritation and vomiting, but in all, or nearly all of which, other remedies were required to aid in restoring a healthy condition to the bowels. In the first stage of active cholera morbus, both in children and adults, we have many times promptly arrested the active symptoms by using the following formula:

R.—Carbolic acid crystals. - - grs. vi.
Glycerine, - - - - - oz. ss.
Camph. tinct. opii, - - - oz. jss.
Water, - - - - . oz. ij.—M.

Give to adults one teaspoonful every half hour or hour until the symptoms are relieved, and doses proportionately less for children. In active dysentery or acute inflammation of any part of the mucus membrane of the alimentary canal, we have found little or no advantage from the use of carbolic acid, but in many cases of chronic dysentery, accompanied by flatulency and gastric irritability, it has afforded much relief when given with paregoric, as in the last formula stated above, and repeated every three, four, or six hours.—*Medical and Surgical Reporter.*

IODIZED MILK.—From Hoffman's most admirable report on the "Progress of Pharmacy, 1869," we make the subjoined extract, which has a practical value for the physician :

*Iodine and Milk.*—It is well known that milk takes up iodine, disguising its taste, smell, and color completely ; since iodine is antiseptic, iodized milk keeps for some time. Dr. Hagar calls attention to this fact, and suggests that this, perhaps, is the mildest form of administering iodine. Its therapeutic effect seems to be equal only to about one-fifth of the iodine.

Hagar thinks iodized milk will soon become a favorite form of administering iodine, and suggests the following mode of preparation : One part of iodine dissolved in ten parts of alcohol, admixed with ninety parts of fresh warm cow's milk.—*Med. Press & Cir.*

A NEW IODINE PAINT.—Dr. J. Warring-Curran recomends (Medical Press and Circular., August 3, 1870,) the following paint as an excellent application in cases of glandular enlargements and scrofulous diseases, wherein iodine is called into requisition. Rub down half an ounce of iodine and a like quantity of iodide of ammonium in a Wedgewood mortar, and gradually dissolve it in twenty ounces of rectified spirit ; to this add four ounces of glycerine, shaking well the solution. Iodide of ammonium, the author thinks, is a more powerful absorbent than iodide of potassium.—*Medical News and Library.*

ACONITE AND MURIATE OF AMMONIA IN NEURALGIA.—In the Chicago Medical Examiner, for November, Dr. J. T. Newman confirms the usefulness of aconite and muriate of ammonia in ovarian neuralgia. The prescription he gives is :

R.—Ammonæ muriatis, - - - dr. ij.
Tr. aconiti, . - - f. dr. ij.
Syrup aurantii cort., - - - f. oz. viij.—M.

S.—Teaspoonful three times a day.

IODINE GARGLE.—M. Cullier (Union Medicale) advocates the following in syphilitic ulceration of the mouth and throat, and in œzena : Iodide potassium 1 part, honey syrup 30, and decoction of barley 120 parts.

DIGITALIS INFUSION.—According to Richelot, (L'Union Med.,) the infusion of digitalis is more effective in dropsy than the decoction. Half-drachm to six ounces of water should be used in twenty-four hours.

SPERMATORRHŒA.—Spermatorhœa has, by most writers, been divided into two forms;—*Tonic* and *Atonic*, or sthenic and asthenic, which require very different modes of treatment.

While the tonic form evinces excitement and irritation, the atonic shows depression and relaxation. Our treatment in the first, or tonic condition, must be antiphlogistic and sedative, and in my hands no single remedy has proved more deserving of confidence than gelseminum, and when exhibited in full doses seldom fails to soon produce a decided improvement. Lupulin and hyoscyamus are also worthy of a trial. Bromide of potassa has, by some, been very successfully employed; and where there is much irritation of the bladder, belladonna and camphor are by no means to be neglected.

In the second, or atonic condition, the treatment should be tonic and stimulating. The tinct. ferri chloride is one of our best medcines here, and when joined with the shower bath, plenty of exercise in the open air, and sleeping on a hard mattress at night, will usually effect a speedy cure.

In certain forms of the disease I have found the following prescription very successful:

R.—Fluid Ext. Gelseminum, - - oz. ii.
Fluid Ext. Ergot, - - - - oz. iss.
Fluid Ext. Hydrastis Canadensis, dr. ij.—M.

Give a teaspoonful every four hours.

But, if the case has long resisted treatment, I use a bougie smeared over with nitrate of silver ointment (5 grains to the ounce of simple cerate), which I introduce into the urethra every third night, letting it remain ten or fifteen minutes, and, if there is much thickening or induration of tissues involved, which is often the case where there is a scrofulous diathesis, I prescribe

R.—Syr. Stillingia Comp., - - oz. jii.
Fluid Ext. Iris Versicolor, - - dr. iv.
Fluid Ext. Helianthemum Canadense, oz. ij.
Potass. Iodid., - - - - dr. i.—M.

Give teaspoonful three times a day. Counter-irritation to the perineum is also a valuable adjunct, and I believe when this line of treatment is properly followed out, cauterization will be found unnecessary.—*University Journal.*

NEW SALVE FOR CHAPPED NIPPLES.—M. Blacquiere has devised the following ointment, which is said to give unfailing relief to cracked nipples, in three or four applications: Cacao butter, 10 parts; sweet almond oil, 2 parts; extract of rhatany, 1 part. Mix.—*Practitioner.*

ANÆSTHETICS.—Dr. S. R. Niasley, (Journal Mat. Med.) says: "I have been in the habit of using the Bisulphide of carbon as a local anæsthetic for several years. I have tested its efficacy and potency in facial neuralgia, hemicrania, ondontalgia, and lumbago, and the speedy relief it afforded to the sufferer was almost instaneous. My mode of application was this: place a pledget of cotton into a large mouth bottle, saturate it well with the Bisulphide and apply it to the painful part, and as soon as the patient complains of smarting sensation, change the bottle, carefully following the course of the principal nerve that seems to be involved in the difficulty. I have used a combination of Rhigolene and the oil of Peppermint as a local anæsthetic in a number of neuralgia cases, that presented themselves at my office for relief, and thus far my success in those cases has been far beyond my most sanguine expectations. After several applications they express themselves cured. I have recently been in the habit of adding an etheral collodion to the compound, and I am gratified to say that in the combination, I have a specific, which will, under almost any circumstances, when the part is accessible, relieve the patient instantaneously; its effects are magical. In a severe case of tic douloureux where the famous "Wolcott Pain Paint" failed to give the patient a moment's relief, this compound was tried and in several minutes the patient was relieved and even expressed herself free from any pain, and the apprehension of a recurrence of the fearful and terrible monster.

POISON OAK.—Dr. J. B. A. Risk, of Morgan, Ky., writing to the Cincinnati Medical Reportory, states that the erysipelatoid affection of the skin induced by any one of the family of Rhus—whether the radicans, toxicodendron, vernix, etc.—speedily subsides under treatment with a decoction of quercus alba. The diseased parts should be in contact with the decoction for 30 or 40 minutes, or longer, and the application should be repeated every four hours.

TREATMENT OF SUN-STROKE.—Sixteen cases of sun-stroke were brought into the Pennsylvania Hospital for treatment, [Reports Vol. 11.] during the heated term of the summer of 1868. The leading symptoms were extreme jactitation, restlessness and convulsions. They were treated by Dr. Hutchinson, with hypodermic injection of one-fourth grain sulph. morph. followed by frictions with ice. All but four recovered. The effect of the morphia was prompt and salutary. In some cases it was necessary to follow up the treatment with brandy, either by mouth or enema.

*New Plan of Dressing Wounds.*—The latest novelty in the mode of dressing wounds following amputation or other causes, is reported to The Lancet by its Paris correspondent. It originated with M. Alphonse Geurin, and consists in introducing cotton-wool into the stump or wound immediately. The amputated limb, to take this example, is then wrapped round with dry cotton-wool, a bandage being then applied, and tightened a little on subsequent days, if necessary, to maintain mild compression. The dressing, however, remains undisturbed until the twentieth or twenty-fifth day. when, on removing the packet of wadding, a glassful of pus is found in the folds of the cotton, and the wound is discovered to be quite healed. Notwithstanding the high mortality which existed during the German seige, M. Guerin obtained six successful results out of nine amputations of the thigh treated with this method, and all of his cases of amputation of the leg did well.

Stimulating Liniment.—Essence of terebinth,
Tincture cantharides, Aq. Amon., aa. gram. xxx.
Tincture opii, - - - - gtts. xx.
For rubbing in paralysis of the extremities.

Bromide of Potassium for the Troubles of Teething.—The bromide of potassium is highly spoken of by Dr. Hoehling (St. Louis Medical Journal), and by Dr. Caro (Medical Record,) as a remedy for faulty nutrition, irritability of the stomach, vomiting, restlessness, diarrhœa, etc., resulting from teething. From half a grain to a grain, given every hour or two, will, in a short time often procure relief and sleep when other means have failed. The following is selected from Dr. Caro's list of twenty cases. S. Fay, nine months old, has six teeth, nursed by a healthy mother. After fifteen days of diarrhœa with from fifteen to twenty passages every twenty-four hours, was cured by a mixture of one drachm of bromide of potassium in an ounce of mucilage, twenty drops every hour. Dr. Caro adds: "I have had fifteen similar cases in every form, having been sick from fifteen to twenty days before coming to me, all treated and cured by the bromide of potassium."

Carbolate of Lime in Pertussis.—Dr. Snow, of Providence, R. I., has suggested the use of carbolate of lime in hooping-cough, and in all cases it has apparently produced a marked effect in diminishing the frequency and severity of the paroxysms. Small quantities of the carbolate of lime are placed in saucers in the room where the child sleeps; merely sufficient to make the odor perceptible.—*Medical Record.*

Bismuth in Dysentery.—The subnitrate of bismuth, in large doses, is highly lauded for the treatment of the dysentery of hot climates, by a French navy surgeon. He gives it in very large doses, beginning with half an ounce a day and rapidly increasing to two ounces and upward daily. In small doses he thinks it not only inefficient but hurtful. During convalescence it must be continued in dimished quantities. After a few doses, the patients were able to take such articles of diet as before the administration of the bismuth they had not dared to touch.

Veratrum Viride in Pericarditis.—Dr. J. Waring-Curran states that he has found this drug of the highest value in the treatment of pericarditis. The extract made by inspissating the juice of the root is the preparation he has invariably employed, prescribing it in two-grain doses, with one grain of calomel in the form of pill, every two hours, and carefully watching its effects. Its power of reducing the frequency of the pulse, and of increasing the renal and hepatic secretions, lead him to regard the veratrum viride as almost a specific for pericarditis. In cases of acute rheumatism in which pericardial symptoms began to be manifest, he feels assured "that the mischief was baffled by early and careful exhibition of ten-drop doses of the tincture of veratrum viride in the asthmatic mixture."

Treatment of Hæmoptysis by Ergot of Rye.—Dr. Horace Dobell, M. D., Senior Physician to the Royal Hospital for diseases of the Chest, (British Medical Journals,) says: "In spite of the fashionable outcry against complicated prescriptions, Dr. Dobell ventures to give the following as the most efficacious, and, as it seems to him, the most rational, combination of remedies for a case of profuse tubercular pulmonary hemorrhage:

R.—Extract ergotæ, liquid drachm, ij (to contract the vessels); tincture digitalis, drachm, ij (to steady the heart); acidi gallici, drachm, j (to clot the blood); magn. sulphatus, drachm, vj (to relieve congestion); acidi sulphurici diluti, drachm, j (to assist the rest); infusi rosæ acidi, ad oz. viij (to make a mixture). A sixth part every three hours till hemorrhage is arrested.

In any given case, either of the ingredients may be omitted, if the symptoms indicate that it is not required, or that it has already done its duty.

Antidote for Poisoning by Phosphorus.—The essence of turpentine is an antidote for poisoning by phosphorus. Let all remember this when a child accidentally helps itself to matches.—*Oregon Medical and Surgical Reporter.*

New Use of Potassium.—Dr. A. De Beaufort (Bullettin de Therapeutique) reflecting that iodide of potassium is freely eliminated in the tears and uterine mucous, tried it in free doses, in cases of chronic inflammation of the lachrymal tube, and also in chronic metritis. His success was most decided. He says: "In cases of internal metritis, with abundant leuchorrhœa, and all that train of circumstances which render so many women miserable, I have often seen, when all other means have failed, prompt and decided amelioration, and in some cases positive cure, result from the free use of iodide of potassium.

To Remove the Bitterness of Sulphate of Magnesia.—To remove the bitterness of sulphate of magnesia, which is the chief drawback of this useful saline aperient, it suffices according to the Bullettin de Therapeutique, to boil a little coffee in the solution of sulphate; the flavor of the coffee masks that of the medicine. The flavor of the decoction of senna may be covered in the same way. —*Medical and Surgical Reporter.*

Better—R.—Sulphate of Magnesia, - - oz. j.
Mint Water, Simple Water, aa. oz. iv.—M.

—*Nashville Journal of Medicine and Surgery.*

Treatment of Typhoid Fever by Glycerine.—Mr. E. Shedd claims (British Medical Journal) great success in the treatment of typhoid fever, by this plan: "As soon as there is any tenderness in the abdomen upon pressure, I prescribe drachm-doses of glycerine (in the case of an adult), to be repeated three times a day. Under this treatment, the temperature gradually subsides, becoming normal towards morning, and rising to 99° Fahr. towards evening. The secretion soon improve; a profuse perspiration frequently prevails; diarrhœa is quickly checked; and the patient becomes convalescent.

"Of the numerous cases which have come before me in my practice, I have treated twenty-seven in the manner described, and with complete success, as not a single death from typhoid fever has occurred."

Puncture of the Intestines, for the Relief of Tympanitis, was spoken of at length in the meeting of the Academy of Sciences, at Paris, on the 10th of July, when, in addition to the cases reported by other physicians, M. Fonsragrives stated that he had already performed the operation eighty times with favorable results, the instrument used being a very fine trocar, and the operation being followed by no more serious symptoms than attend the tapping of a hydrocele.—*Medical Record.*

Iodoform as a Neurotic.—Dr. Ephraim Cutter, of Boston (Gynæcological Society Journal., "The Doctor,") "gave iodoform to a lady of about twenty-five years, who had been suffering for years under a chronic indurative enlargement of the cervix uteri,—pronounced cancerous by some physicians. No ulceration, but much pain in the cauda equina and through both thighs. This was constant, worse at night, and severe. Various anodynes and sedatives not giving much relief, Dr. Cutter tried the iodoform and iron, one grain pill, sugar-coated, three times a day, with an immediate and powerful effect. The patient complained of indescribable feelings in her stomach, which annoyed her; but there was a cesation of the pain. On taking one pill a day, the iodoform was well borne, with marked alleviation. A vaginal suppository of iodoform two grains, cocoa butter and white wax enough to make a weight of thirty grains, was then given at night with similar results. There was not a cure of the enlargement, but the patient was relieved, and now by the occasional use of the iodoform, she manages to get along better than ever before. It acts as a calmant, sedative anodyne. It is bland and unirritating. Its ordor is its great objection.

In an old subject of asthma, who suffered much from hyperæsthesia of the skin, forming a pure neurotic affection, the iodoform and iron pill, given thrice daily, gave relief, but did not cure. It acted promptly, and, by taking occasional doses, the lady is comfortable. It also quieted the asthmatic symptoms. In other ladies exquisite pain was at once relieved by the same local use of the iodoform.

A young man had a bleeding, irritable ulcer of the leg, about two inches in diameter, and situated just above the external malleolus of the leg. The hæmorrhage was considerable, and the pain so severe as to deprive him of sleep. The hæmorrhage was checked by the persulphate of iron. Sulphate of morphia, gr. j.; dr. j. laud., was applied to the surface, and bandages put on. Opium was administered at night, and a comparative degree of comfort was attained. On applying iodoform, grs. xv. to dr. j. laud., to the surface night and morning, the pain entirely ceased; and in three days the healing process was so rapid that more than one-half of the sore was covered by healthy cicatrix. Dr. C. exclaims "I was astonished, and could hardly credit it; but this did not alter the fact." In another case of irritable ulcers of the leg, the application of iodoform was followed by relief of the pain, but not by healing."

As general and local anæsthetic, anti-spasmodic and resolvent, we have repeatedly employed iodoform with great advantage.—*Medical Cosmos.*

LYCOPERDON OR PUFF BALL AS A STYPTIC AND ANÆSTHETIC.—C. Brewster, L. D. S., Montreal, (Canada Journal Dental Science,) invites attention to the value of this agent, and states that after thirteen years experience, he regards it the best of all known local styptics. Dr. W. G. Beers, the editor of this journal, testifies to the same effect, and thinks it "the simplest and speediest styptic to be had." He says: "We know from experience of its success in arresting alveolar hæmorrhage where the actual cautery, perchloride of iron, and all other means failed. Only one case (in the Medical Gazette of 1842,) is cited where it seemed to fail. About a month ago, I used it in a dangerous case, which had resisted all other treatment, and it instantly arrested the hæmorrhage.

Drs. B. W. Richardson and Snow, made themselves insensible by its fumes, and the former narcotized over a thousand animals with its vapor, and operated successfully; the effect lasting, in many cases, for half an hour. Dr. Richardson frequently inhaled the fumes through a hookah pipe, letting them pass first through potash water to clear them of carbonic acid. He recommended a slight inhalation from a common pipe, as a local anæsthetic or temporary relief, to those suffering from the pain of an exposed pulp.

Those who have not used it ought to have it on hand. Large quantities are found down about Murray Bay, and throughout the Eastern Townships of the Province of Quebec, and, indeed, in the vicinity of most dry and sandy soils."—*Med. Cosmos.*

ETHER FOR TAPEWORM.—Dr. Lurtel (Gazette Medicule de Paris) has tried with success, the following method: He gives in one dose two-thirds of an ounce of ether, followed two hours afterwards, by an ounce of castor oil. The worm is discharged entire or almost so, and always with the head intact. No pain is caused by this teatment.—*Rodolphe.*

ERGOTIN SUBCUTANEOUSLY IN METRORRHAGIA.—In the Presse Med., there is the report of a case in which the bleeding, whose cause was obscure, had continued for two months, being occasionally checked for a few hours at a time, by cold injections, and ergot internally. Ergotin to the amount of 1-12 grm., dissolved in a mixture of glycerine and distislled water was injected subcutaneously, and less than four hours after the discharge ceased. As a measure of precaution, the injections were repeated on the three following days, and there was no return of the hæmorrhage. —*Medical Press and Circular.*

Columbo and Phosphate of Lime in Vomiting of Prenancy. Dr. E. J. Beall writes (Med. Archives,) in reference to the value of these agents in symphetic vomiting of pregnancy: "Let me add my testimony as to the beneficial effects of the Columbo, predicated upon long and frequent use in combination with Phosphate of Lime, and for brevity, offering no solution as to its physiological or therapeutic effects. As far back as 1854, I prescribed an infusion of Columbo Root and Phosphate of Lime for Mrs. Dr. Lewis, of Marshall, Texas, for the condition above mentioned, with good results, and since in divers cases. I commend the combination to the profession as worthy of trial.—*Medical Cosmos.*

Burns and Scalds.—I have treated a good many cases of burns and scalds, and to my entire satisfaction. I dissolve white lead in flax seed oil, to the consistency of milk, and apply over the entire burn or scald every five minutes. I have been in the habit of using a soft feather to apply the liniment. I have used this preparation a great many times in the fifteen years of my practice, and have never been disappointed; it gives relief the soonest and is more permanent in its effects than any preparation I am acquainted with.

I think that any one testing it wiil be satisfied. It should be applied often, and a full dose of an opiate will be advantageous if the burn is deep.—*Dr. Waller.*

Nux Vomica in Colic.—Dr. C. J. Cleborne states, (Medical Record): "In cases of simple uncomplicated colic I know of no remedy so effectual and so speedy in giving relief as the tincture of nux vomica, in two drop doses every hour. Rarely more than two doses are required, when aided by a warm bath, or a hot bran poultice over the abdomen."—*Med. Cosmos.*

Treatment of Night-Sweats in Consumption.—Powdered borax, dr. vss., washed sulphur, oz. i., sub-nitrate of bismuth, drs. iss.; divide into forty powders, one to be given every two hours (twelve a day). Four to five days of treatment will suspend or diminish this troublesome and exhausting symptom, and give much relief to the patient.—*Rodolphe.*

Oil of Erigon in Cystirrhœa.—Dr. M P. Shelton, Virginia, (Jour. Mat. Med.) says: "Having a very obstinate case of cystirrhœa, which had resisted all other remedies, I concluded to try the Erigeron. It was a perfect success. Not quite an ounce was taken before the cure was complete." This was a case of several years standing.

*Cod-Liver Oil in Eczema.*—A highly respectable medical gentleman, of Charlestown, Mass., (Boston Journal of Chemistry,) advocates the use of cod-liver oil in eczema, and notes the case of a child, twenty-two moths old, who had been afflicted with the affection for nineteen months. Its entire body was covered with the eruption. The use of the oil was suggested, and the child is now nearly well; scarcely a trace of the disease remains. This has been accomplished during dentition.

GLYCERINE CREAM.—This recipe is excellent for chapped lips:

Take of Spermaceti, four drachms.
White wax, one drachm.
Oil of almonds, two troy-ounces.
Glycerine, one troy-ounce.

Melt the spermaceti, wax, and oil together, and when cooling stir in the glycerine and perfume.

TURPENTINE AS A PREVENTIVE OF CHOLERA.—Dr. E. J. Beall, (Medical Archives,) says: "During the year 1866, when cholera was epidemically prevalent at Houston, Millican and San Antonio, Texas, I had occasion to treat seventy-odd cases on two farms in Brazos county. Simultaneously, with the use of sulph. iron in solution to inside of building, lime to outside, etc., I directed each person—two hundred, perhaps—to carry, and distributed to that effect (as also using it myself), vials of oil of turpentine, to be applied to moustache, whiskers, shirt bosom, etc., when the parties were in the neighborhood of the effected cabins. No cases occurred with those resorting to this prophylactic. Under treatment followed, eleven deaths occurred of the number stated, several of which were so rapid as to haye received no treatment. The first case that occurred was with a wagoner, who had been to Milican, and was taken just before reaching home. His family, consisting of wife and children, were the next cases on the place. The wind coming from the west, affected next two cabins east. Two days thereafter the wind getting from the north, the cabins immediately south were affected. Visitors from an adjoining farm transferred the disease to that place—seventy cases, with the mortality mentioned, being the result."—*Medical Cosmos.*

ACUTE MANIA.—Speaking of the value of certain drugs in insanity, Dr. Clouston, says: "A mixture of one drachm of bromide of potassium with one drachm of the tincture of cannabis Indica is more powerful to allay excitement than any of the other drugs or stimulants tried. It is more uniform and certain in its effects, more lasting, interferes less with the appetite; and to produce the same effect the dose does not require to be increased after long continued use.

Single doses of opium tended to raise the temperature and to lower the pulse; single doses of the mixture above-mentioned to lower the temperature and quicken and weaken the pulse, of bromide of potassium alone to raise the temperature and lower the pulse, of cannabis Indica alone to raise the temperature and quicken the pulse, of whiskey to lower the temperature very much and slightly to quicken the pulse, and of beef tea to lower the temperature in the least degree and to lower and strengthen the pulse.

By giving bromide of potassium and cannabis Indica together, not only is the effect of either given separately immensely increased, but the combination has an essentially different action from either of them given alone.

Bromide of potassium alone can subdue the most violent maniacal excitement, but only when given in immense and dangerous quantities, and its effects are so cumulative while so given, that after they have once begun to appear they increase for days after the medicine has been stopped, almost paralysing the cerebrum and sympathetic.

TREATMENT OF GONORRHŒA.—M. Cullerier gives for the aborative treatment of gonorrhœa, large doses of copaiba—from four to five drachms a day. This is continued for four or five days after the discharge disappears. When the inflammatory stage is over, he uses the following injection to complete the cure:

R.—Sulphate of Zinc, Sugar of Lead, aa. gr. xv.
Water, - - - - - f. oz. iv.—M.

S.—Inject twice daily.

Or, Alum, - - - - - dr. ss.
Water, - - - - f. oz. iv.—M.

S.—Use an injection twice daily.—*Baltimore Med. Bulletin.*

QUINIA IN HAY ASTHMA.—It is stated: "Prof. Helmholtz, who had yearly, for twenty years, been a sufferer from hay fever, discovered vibrios of a peculiar character in the secretions of his nose. After reading the experiments of Dr. Bintz, on the power of quinia over these lower organisms, he injected into his nostrils a weak solution of quinia, and the symptoms disappeared. The next year, as soon as the symptoms reappeared, he again used the same injection, and with equal success."—*Med. Cosmos.*

Formulæ for Dyspepsia.—Dr. Charles Carter, Philadelphia, writes (Indiana Jour. of Med.), "In the extensive class of cases of the latter disease (Dyspepsia) met with in *Dyspensary* practice, having similiar general symptoms, and with evidence that the whole alimentary tract is in an abnormal state, I have found either of the following combinations of Gentian very serviceable :

| | | |
|---|---|---|
| R.—Gentian Root, (broken), | - | dr. vi. |
| Wild Cerry Bark, | - - - | oz. ss. |
| Quassia, | - - - | dr. ii. |

Infuse in two pints of hot water. Dose, a wineglassful before meals.

| | | |
|---|---|---|
| R.—Sub-Nit. Bismuth, | - - | dr. i. |
| Calc Carb. Precip., | - - - | dr. iii. |
| Fl'd Ext. Gentian, | - - | oz. ss. |
| Aqua Cinnamon, | - - | oz. iiiss. |

Sig.—A teaspoonful before and after each meal.

This latter is not a very elegant mixture, for the powders which must be given suspended in the liquid, precipitate. The mixture therefore, requires to be well shaken before using. It is, however, very effective. I have recently administered it in a very severe and protracted case of dyspepsia, with complete relief of all the symptoms.

*Digitalis in Cardiac Diseases.*—Dr. A. L. Loomis reported to the New York Pathological Society (Medical Record,) several interesting cases of cardiac diseases. In alluding to "these cases of obstructive diseases," Dr. C. C. Lee, said: "In view of the acknowledged therapeutic value of digitalis, he thought it strange that the remedy should not be more extensively used. He had not unfrequently met with cases in which it had not been employd at all.

Dr. Loomis said that this latter was in a measure explained by the want of reliability in the drug. As usually found in the market, it was good for nothing. He was in the habit of relying entirely upon the infusion made from the fresh English leaves (oz. ss. Oj.). There was an exception to its universal use in cardiac disease, and that was in cases of pure hypertrophy without dilatation.

---

Croup.—The first account of this disease in the English language, by this name, is to be found in a letter from Dr. Patrick Blair, of Cupar Angus, to the celebrated Dr. Mead, of London, dated July 6th, 1713.

Phytolacca Decandra in Cancer.—Dr. O. Cock, Dayton, Ohio, says (Med. and Surg. Reporter): "With the steady and persistent administration of the phytolacca decandra, in the first stages of cancer, he has in many cases seen the tumor absorbed, and a cure effected without the knife or escharotics.

His method of preparing and using the remedy, is as follows:

| | |
|---|---|
| R.—Phytolaccæ Radicis (Poke Root), | oz. ij. |
| Alcohol dilute, or pure whisky, - | Oj. |

Let it stand 14 days and filter.

Of this the patient may take, after eating, from 15 drops to a tablespoonful, as will be borne by the stomach. He derived the most benefit from its use when pushed until its emetic effect is produced, and then continued the dose that the stomach would bear without excessive nausea. The effect of the medicine will be apparent in the space of two weeks—first in relieving the pain so significant of cancer, and in arresting the growth of the tumor, and in the general improvement of the health of the patient.

If, after administering the medicine for a few weeks, its beneficial effects are not manifest, it will be well to desist for a short time and then commence as at first, when better effects will be produced. He has obtained favorable results from the use of injections of the tinct. iodine, from five drops to a drachm, and the subsequent application of the pulverized or grated Poke root, in the form of a poultice, intermitted with the use of emollient poultices of carrots, or bread and milk.

Pomatum for Chapped Lips.—The following is from the Drnggist's Circular:

| | |
|---|---|
| R.—Lard, . . . . . . | 16 parts. |
| Cacao oil, . . . . . | 24 " |
| Spermaceti, . . . . . | 8 " |
| Yellow wax, . . . . | 3 " |
| Alcanna root, . . . . | 1 " |

The substances are fused for a quarter of an hour at a gentle heat, then strained through a cloth and mixed with—

| | |
|---|---|
| Oil of lemon, | |
| Oil of bergamot, aa . . . | 1-16 part. |
| Oil of bitter almonds, . . | 1-15 " |

when the mass is poured into suitable vessels to cool.

